# *CYP2B6* Non-Coding Variation Associated with Smoking Cessation Is Also Associated with Differences in Allelic Expression, Splicing, and Nicotine Metabolism Independent of Common Amino-Acid Changes

**DOI:** 10.1371/journal.pone.0079700

**Published:** 2013-11-15

**Authors:** A. Joseph Bloom, Maribel Martinez, Li-Shiun Chen, Laura J. Bierut, Sharon E. Murphy, Alison Goate

**Affiliations:** 1 Department of Psychiatry, Washington University School of Medicine, St. Louis, Missouri, United States of America; 2 Department of Biochemistry Molecular Biology and BioPhysics, University of Minnesota, Minneapolis, Minnesota, United States of America; University of Kentucky, United States of America

## Abstract

The Cytochrome P450 2B6 (CYP2B6) enzyme makes a small contribution to hepatic nicotine metabolism relative to CYP2A6, but CYP2B6 is the primary enzyme responsible for metabolism of the smoking cessation drug bupropion. Using *CYP2A6* genotype as a covariate, we find that a non-coding polymorphism in *CYP2B6* previously associated with smoking cessation (rs8109525) is also significantly associated with nicotine metabolism. The association is independent of the well-studied non-synonymous variants rs3211371, rs3745274, and rs2279343 (CYP2B6*5 and *6). Expression studies demonstrate that rs8109525 is also associated with differences in *CYP2B6* mRNA expression in liver biopsy samples. Splicing assays demonstrate that specific splice forms of *CYP2B6* are associated with haplotypes defined by variants including rs3745274 and rs8109525. These results indicate differences in mRNA expression and splicing as potential molecular mechanisms by which non-coding variation in *CYP2B6* may affect enzymatic activity leading to differences in metabolism and smoking cessation.

## Introduction

Tobacco use remains the largest cause of preventable mortality worldwide and improvements in smoking cessation treatments have great potential to impact both public health and individual quality of life. Smoking-related phenotypes are highly heritable [1]–[3]; genetic studies therefore provide a powerful tool to reveal the biology underlying smoking behavior and dependence. Single nucleotide polymorphisms (SNPs) near the *CYP2A6* nicotine metabolism gene were among the few loci associated with consumption of cigarettes per day (CPD) with genome-wide significance [4], [5]. We have since determined that these SNPs are proxies for several functionally important *CYP2A6* haplotypes [6]. Such synthetic associations, resulting from the coincidental linkage of common markers with multiple less-frequent causal variants, have been proposed as sources of unexplained GWAS findings [7]. With this in mind, we embarked on a study of the nearby and similarly complex *CYP2B6* locus. CYP2B6 plays a small role in nicotine metabolism [8], [9] but is the primary enzyme responsible for the metabolism of substrates including methadone, efavirenz, cyclophosphamide, and bupropion [10], a drug prescribed for smoking cessation. A candidate gene association study recently reported a non-coding polymorphism in *CYP2B6* (rs8109525) associated with smoking cessation, both with and without bupropion treatment [11]. Other studies have reported a potential link between the *CYP2A6* gene and smoking cessation [12], [13]. We therefore sought to determine whether *CYP2A6* and nicotine metabolism might contribute to the reported association [11] with SNPs in the adjacent *CYP2B6* locus.

However, contrary to our expectations, the data presented in this study indicate that rs8109525 and other closely linked SNPs are significantly associated with nicotine metabolism independent of *CYP2A6* genotype. Furthermore, we find that rs8109525 is significantly associated with hepatic *CYP2B6* expression. Importantly, both associations are independent of the well-studied *CYP2B6* alleles, *5 (rs3211371) and *6 (rs3745274/rs2279343), which are defined by common amino acid changes associated with altered metabolism of other substrates. Complicating these results, *CYP2B6* variants including rs8109525 and rs3745274/rs2279343 are also shown to be associated with aberrant *CYP2B6* mRNA splicing. Splicing of mRNA is a key regulatory point for gene expression (reviewed in [14]). Rare variants that disrupt splicing or alter the inclusion of both constitutive and alternatively-spliced exons have been associated with disease [15]–[23]. Common alleles that alter splicing provide a portion of the genetically determined variance in clinically-relevant traits including nicotine metabolism [24]. The importance of maintaining the balance of exon splicing enhancer and suppressor motifs is demonstrated by the relative infrequency of SNPs that disrupt these motifs, especially near exon boundaries [25], [26]. Aberrant *CYP2B6* mRNA splicing is common and diverse [27], [28]. Here we demonstrate associations between genetic variation and *CYP2B6* mRNA splicing involving many aberrant forms; together with differences in allelic expression, variation in splicing may provide a mechanism underlying common functional differences between *CYP2B6* haplotypes.

## Materials and Methods

This study complies with the Code of Ethics of the World Medical Association and obtained written informed consent from participants. The Human Studies Committee at the Washington University School of Medicine in Saint Louis approved the study. The approval number for the Collaborative Genetic Study of Nicotine Dependence (COGEND) is 00-0203. Participant recruitment from COGEND [29], nicotine metabolism measures and *CYP2A6* genotyping in 189 European Americans were previously described [6], [30] (Table S1). Application of the predictive model of CYP2A6 activity was previously described [30]. Briefly, all analyses of measured metabolism were linear regression analyses performed on a metabolism metric, the ratio of deuterated (D_2_)-cotinine/(D_2_-cotinine+ D_2_-nicotine), determined 30 minutes following oral administration of D_2_-nicotine. The original model parameters were derived from the regression, log (1 – metric)  =  log(α) + log(βH1) + log(βH2) where α is the intercept, βH1 represents the first *CYP2A6* haplotype and βH2 represents the second *CYP2A6* haplotype for each subject. For subjects of European descent, the metric can be determined from genotype based on six SNPs and *CYP2A6* gene copy number as described in table S2. Statistical analyses were performed using the software package ‘R’ (R Foundation for Statistical Computing, Vienna, Austria). All t-tests performed were two-sided.

### Genotyping and Haplotype Determination

*CYP2A6* and *CYP2B6* nomenclature follows official recommendations (http://www.cypalleles.ki.se) except that CYP2A6*1A is defined by the A allele of rs1137115 throughout. rs1808682 genotype was previously determined using a custom designed array as part of a larger study [29]. Genotyping of additional *CYP2B6* SNPs (Table 1) was performed using the KBioscience Competitive Allele Specific PCR genotyping system (KASPar, KBioscience, Hoddesdon, Herts, UK) following standard procedures with custom designed primers (Table S3). KASPar assays were set up as 8μl reactions and measured with the 7900HT Fast Real Time PCR System (Applied Biosytems, Foster City, CA, USA). *CYP2B6* haplotypes were determined using PHASE version 2.1.1 [31], [32]. Linkage disequilibrium was determined using Haploview [33].

**Table 1:** *CYP2B6* haplotypes in metabolism experiment.

Haplotype name	rs1808682; –7763	rs8109525; –5293	rs34223104; –82 (TATA box)	rs8192709; 64 (R22C)	rs35303484; 136 (M46V)	rs8100458 (1st intron)	rs36060847; 12820 (G99E)	rs12721655; 13072 (K139E)	rs3745274; 15631 (Q172H)	rs2279343; 18053 (K262R)	rs28399499; 21011 (I328T)	rs35979566; 21388 (I391N)	rs3211371; 25505 (R487C)	Alleles	Frequency (%)
*1A	G	A	T	C	A	T	G	A	G	A	T	T	C	63	16.2
*1A	A	A	T	C	A	T	G	A	G	A	T	T	C	24	6.2
*1H/J	G	G	T	C	A	C	G	A	G	A	T	T	C	67	17.3
*1H/J	A	G	T	C	A	C	G	A	G	A	T	T	C	48	12.4
*1H/J	G	A	T	C	A	C	G	A	G	A	T	T	C	1	0.3
*1H/J	A	A	T	C	A	C	G	A	G	A	T	T	C	1	0.3
*2	G	A	T	**T**	A	T	G	A	G	A	T	T	C	15	3.9
*2	G	G	T	**T**	A	C	G	A	G	A	T	T	C	2	0.5
*2	A	A	T	**T**	A	T	G	A	G	A	T	T	C	1	0.3
*4	G	A	T	C	A	T	G	A	G	**G**	T	T	C	3	0.8
*4B	G	G	T	C	A	C	G	A	G	**G**	T	T	C	2	0.5
*5A	G	A	T	C	A	T	G	A	G	A	T	T	**T**	42	10.8
*5A	A	A	T	C	A	T	G	A	G	A	T	T	**T**	7	1.8
*5B	G	G	T	C	A	C	G	A	G	A	T	T	**T**	3	0.8
*6	G	A	T	C	A	T	G	A	**T**	**G**	T	T	C	75	19.3
*6	A	A	T	C	A	T	G	A	**T**	**G**	T	T	C	18	4.6
*6	G	G	T	C	A	C	G	A	**T**	**G**	T	T	C	2	0.5
*9	G	A	T	C	A	T	G	A	**T**	A	T	T	C	5	1.3
*11	G	A	T	C	A	T	**A**	A	G	A	T	T	C	1	0.3
*12	G	G	T	C	**G**	C	G	A	G	A	T	T	C	2	0.5
*15	G	G	T	C	A	C	G	A	G	A	T	**A**	C	2	0.5
*18	G	A	T	C	A	T	G	A	**T**	A	**C**	T	C	1	0.3
*22	A	A	**C**	C	A	T	G	A	G	A	T	T	C	2	0.5
*22	G	A	**C**	C	A	T	G	A	G	A	T	T	C	1	0.3

Polymorphic sites analyzed are given at the top of each column by rs number, gene position and further relevant description. Haplotypes are ordered by common allele name and frequency in the COGEND metabolism dataset.

### Allelic expression study

DNA and RNA extracted from ninety-nine de-identified normal liver biopsy samples from patients of European descent were supplied by the Tissue Procurement Core, Laboratory for Translational Pathology at the Siteman Cancer Center, Washington University Medical Center. cDNA was prepared from total RNA using the Applied Biosystem High Capacity cDNA Reverse Transcription Kit. cDNA and genomic DNA (gDNAs) from heterozygotes for the assayed SNPs were arrayed together in the same 384-well plates in triplicate, and were run on an ABI-7900 real-time PCR system under standard conditions with assays for rs3211371 (C_30634242_40, Applied Biosystems) and a custom designed assay for rs2279343 (AHBJ12M, Applied Biosystems). The relative expression of both alleles for each expression marker was determined by subtracting the smaller Ct value of one allele PCR reaction from the larger Ct value of the other allele PCR reaction (ΔCt). For the statistical analysis, ΔCt values were obtained as an average of two or three reactions for each sample and data point. Allelic ratios for cDNA were normalized against the average ratio obtained from gDNA for each genotype and marker SNP. For the rs3211371 assay mean gDNA ΔCts for rs8109525/rs8100458 heterozygotes and homozygotes were –0.98±0.06 and –0.88±0.09 respectively. For the rs2279343 assay mean gDNA ΔCts for rs8109525/rs8100458 heterozygotes and homozygotes were –2.31±0.08 and –2.33±0.07 respectively. By comparison, mean cDNA ΔCts for rs8109525/rs8100458 heterozygotes and homozygotes were 0.11±0.13 versus –0.36±0.38 for the rs3211371 assay and –0.26±0.45 versus 1.27±0.51 for the rs2279343 assay.

### Quantitative Real-time splice-form expression study

PCR products of the correct size were confirmed for all primer pairs (primer sequences, Table S4) by agarose gel electrophoresis (Figure S1). Reactions for pairs of assays to be compared in each experiment were arrayed together in the same 384-well plate in duplicate pairs, and run on an ABI-7900 real-time PCR system under standard conditions. 10μl reactions included 2x PerfeCTa SYBR Green FastMix ROX (Quant Biosciences Inc., Gaithersburg MD, USA), 0.5 µM each forward and reverse primer, and 1 μl cDNA. Dissociation curves for all primer pairs demonstrated single peaks consistent with little contamination from primer-dimers. Ct values were obtained as the average of two reactions for each sample and assay. The difference in relative quantity detected by each assay was determined by subtracting the smaller average Ct value of one reaction from the larger average Ct value of the other reaction.

## Results

### *CYP2B6* polymorphisms and haplotypes associated with the ratio of nicotine metabolized to cotinine

**Figure 1:**
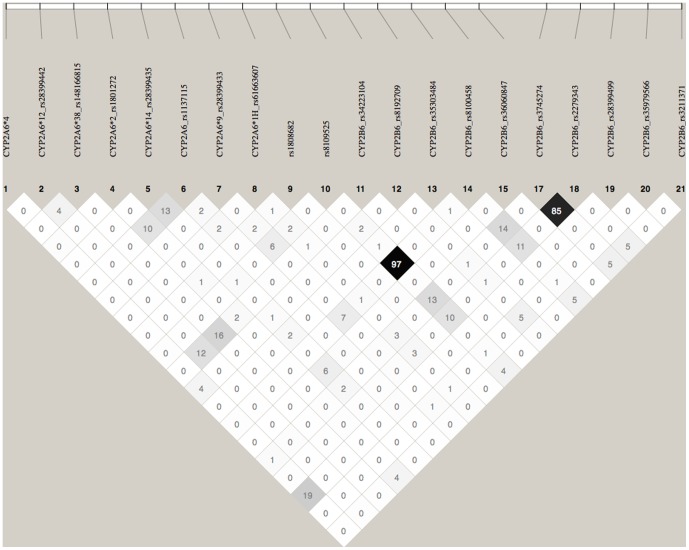
LD structure of the *CYP2A6/CYP2B6* locus for common SNPs genotyped in the COGEND metabolism study. Numbers refer to pairwise R^2^.

We initially investigated two SNPs, rs8109525, located 5 kilobases 5’ of *CYP2B6,* and rs8100458, in the first intron of *CYP2B6,* which is in high linkage disequilibrium (R^2^>0.95) with rs8109525. rs8109525 demonstrated the most significant association with continuous abstinence at weeks 9–12 of treatment in a previous study [11]. Although neither SNP is in high linkage disequilibrium with any of the key *CYP2A6* polymorphisms (Figure 1), both are associated with a small influence upon nicotine metabolism in this data set (rs8109525 p =  0.041, rs8100458 p =  0.024). Both SNP associations with nicotine metabolism remain significant and even improve after inclusion in multivariate regression analyses with *CYP2A6* haplotype variables (rs8109525 p =  0.012 or rs8100458 p =  0.0086), demonstrating that these associations are independent of known *CYP2A6* variants. Previous studies have found that the effect of *CYP2B6* genotype on nicotine clearance is most prominent among subjects with slower metabolizer *CYP2A6* genotypes [34]. Consistent with this hypothesis, we find that the effect is greater among subjects with slower metabolizing *CYP2A6* genotypes (parameter estimate = 0.056, p = 0.002 among n = 34 carriers of CYP2A6*2,*4,*9,*12 and *38 alleles), than among subjects with fast metabolizing genotypes (parameter estimate = 0.011, p = 0.087 among n = 97 subjects excluding CYP2A6*1A,*2,*4,*9,*12 and *38 carriers).

We also genotyped rs1808682, a SNP >7kb 5′ of *CYP2B6* which was reported to be significantly associated with continuous abstinence at weeks 9–52 of smoking cessation treatment [11]. rs1808682 is also associated with nicotine metabolism (p = 0.040), but this association does not remain significant (p = 0.23) in the multivariate analysis including *CYP2A6* haplotypes.

Three non-synonymous SNPs in *CYP2B6* which define the common haplotypes CYP2B6*5 and *6 (rs3211371, rs3745274, and rs2279343, Table 1) are in strong disequilibrium with rs8109525 and rs8100458 (D′>0.8, R^2^<0.15, Figure 1). We hypothesized that one or more of these variants might be responsible for, or confound, the significant associations observed between the non-coding SNPs and nicotine metabolism. To refine the association by creating more complete *CYP2B6* haplotypes, we genotyped these SNPs (rs3211371, rs3745274, and rs2279343), along with the other common (>2% frequency) non-synonymous SNPs in *CYP2B6*, and six rarer SNPs previously shown to affect *CYP2B6* function [35] (Table 1).

Contrary to our expectation, haplotypes CYP2B*5 and *6 did not explain the association between nicotine metabolism and rs8109525/rs8100458. In multivariate analyses including *CYP2A6* haplotypes, CYP2B6*5, CYP2B6*6, and the rs8109525 or rs8100458 major allele reference haplotypes (CYP2B6*1A and *2), all alleles remained independently associated with faster metabolism (Table 2). Multivariate analyses with *CYP2A6* haplotypes and rs8109525 or rs8100458 minor allele reference haplotypes were also significantly associated with metabolism (rs8109525 p =  0.0041, rs8100458C p =  0.0068), providing further evidence that non-coding variants significantly influence *CYP2B6* function.

**Table 2:** *CYP2B6* haplotypes associated with D_2_cotinine/(D_2_cotinine+ D_2_nicotine) in a multivariate regression model with *CYP2A6* haplotypes.

Variable	n	Parameter estimate	Standard error	p^a^
intercept		0.87	0.01	<10^−16^
CYP2A6*1A	56	–0.05	0.01	1.2×10^−7^
CYP2A6*2	12	–0.18	0.02	<10^−16^
CYP2A6*4	6	–0.21	0.03	3.1×10^−14^
CYP2A6*9	23	–0.06	0.01	6.9×10^−6^
CYP2A6*12	8	–0.16	0.02	8.0×10^−12^
CYP2A6*38	2	–0.23	0.04	6.2×10^−7^
CYP2B6*1/2 rs8109525A	105	0.02	0.01	0.015
CYP2B6*5	52	0.02	0.01	0.015
CYP2B6*6	95	0.02	0.01	0.036

a the probability that the parameter estimate is different by chance from the reference, i.e. all *CYP2A6* (n = 271) and *CYP2B6* (n = 126) alleles not included in the multivariate regression. All *CYP2A6* alleles presented are loss-of-function alleles associated with reduced metabolism (−) relative to the reference *CYP2A6* alleles; all *CYP2B6* alleles presented are associated with increased metabolism (+) relative to the reference (rs8109525G) *CYP2B6* alleles. rs8109525 is in high linkage disequilibrium with rs8100458 (R^2^>0.95).

Because rs8109525, rs8100458 or other closely linked SNPs were not associated with any demonstrated or predicted effects on gene function, we also pursued an unbiased approach to identify further SNPs or haplotypes associated with CYP2B6 activity by repeating the multivariate analysis including *CYP2A6* haplotypes in combination with sixteen SNPs across the *CYP2B6* locus previously genotyped in this sample [29]. Among these, the SNP most significantly associated with nicotine metabolism was rs3786552 (p = 0.0061) a variant in the eighth intron in linkage disequilibrium (LD) with rs8109525 (R^2^ = 0.59, D′ = 0.80). The two SNPs are not independently statistically significantly associated with metabolism.

In summary, these data demonstrate that *CYP2B6* haplotypes, defined by non-coding variants, are associated with differences in nicotine metabolism independent of *CYP2A6* genotype. By contrast, we do not detect differences in nicotine metabolism associated with the common amino acid changes that define the CYP2B6*5 and CYP2B6*6 alleles.

### *CYP2B6* variants associated with nicotine metabolism are associated with gene expression

Because the association between non-coding SNPs in the *CYP2B6* locus and nicotine metabolism was independent of known linked non-synonymous SNPs, we hypothesized that the effect might be due to mechanisms other than altered protein function. rs8100458 is in perfect linkage disequilibrium (R^2^ = 1) with rs7254579 (–2320t>c) [36], a polymorphism predicted to disrupt a GATA transcription factor binding site [37] which tags both of the common *CYP2B6* reference haplotypes *1A and *1H/J (Table 1). To determine whether the genetic variants associated with nicotine metabolism were also associated with differences in gene expression we measured allelic expression using allele-specific assays in liver cDNAs from heterozygous individuals. An advantage of this approach is that it avoids confounding factors associated with total expression such as tissue sample quality, diet or disease, and therefore allows differences between alleles to be demonstrated in relatively small numbers of subjects heterozygous for assayable coding SNPs.

**Figure 2:**
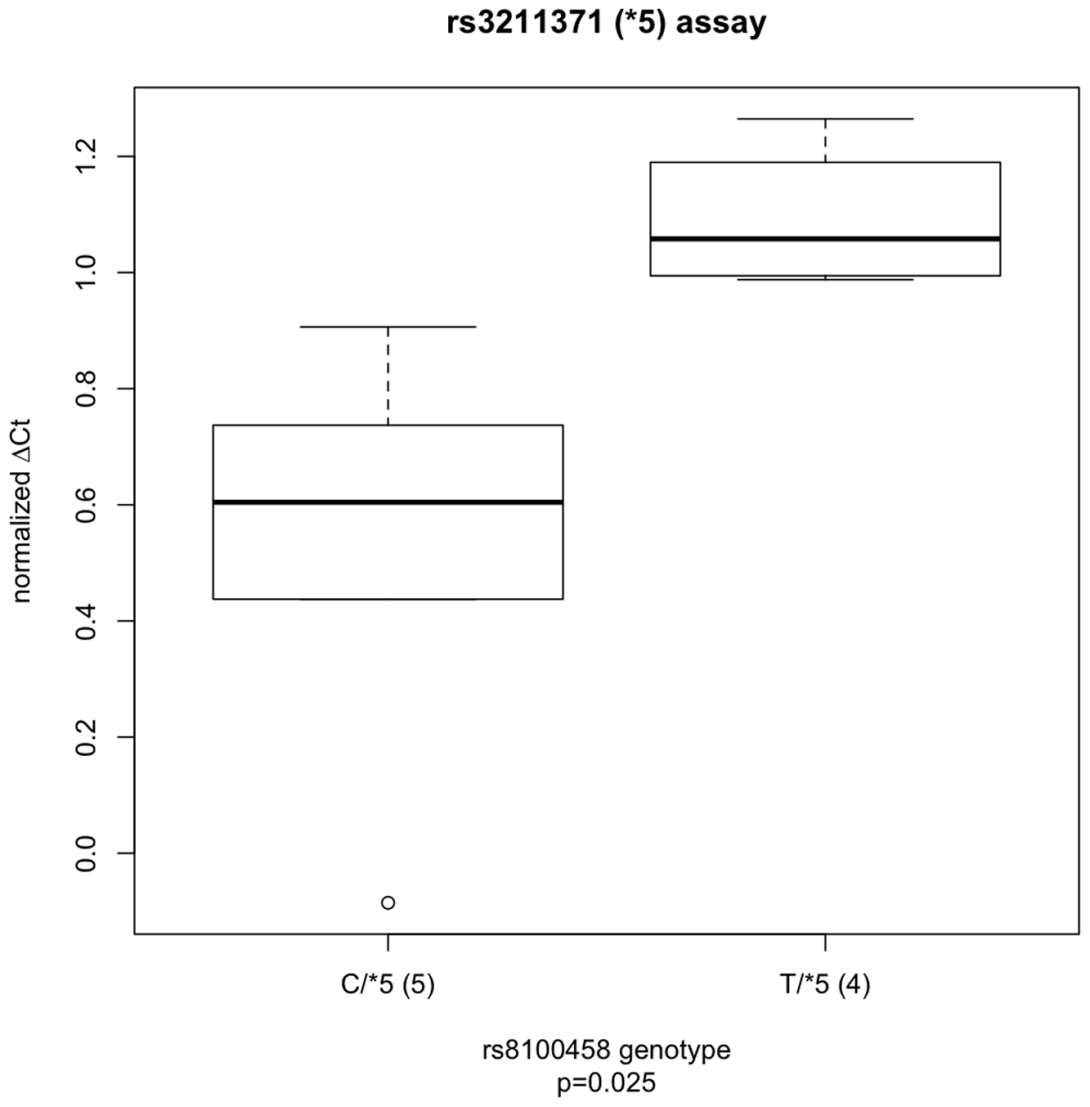
The ratio of allele-specific gene expression (ΔCt) for cDNAs from rs3211371 (CYP2B6*5) heterozygous liver biopsy samples. Samples, excluding rs2279343 heterozygotes, are either heterozygous (CT) at rs8100458 (C/*5, n = 5), or rs8100458 TT homozygotes (T/*5, n = 4). ΔCt differs significantly by genotype (p = 0.025). Relative expression, ΔCt, was determined by subtracting the smaller Ct value of one allele PCR reaction from the larger Ct value of the other allele PCR reaction normalized against the average ratio obtained from gDNAs for each genotype. The boxplot provides a summary of the data distribution. The box represents the interquartile range, which includes 50% of values. The line across the box indicates the median. The whisker lines extend to the highest and lowest values that are within 1.5x the interquartile range. Further outliers are marked with circles.

**Figure 3:**
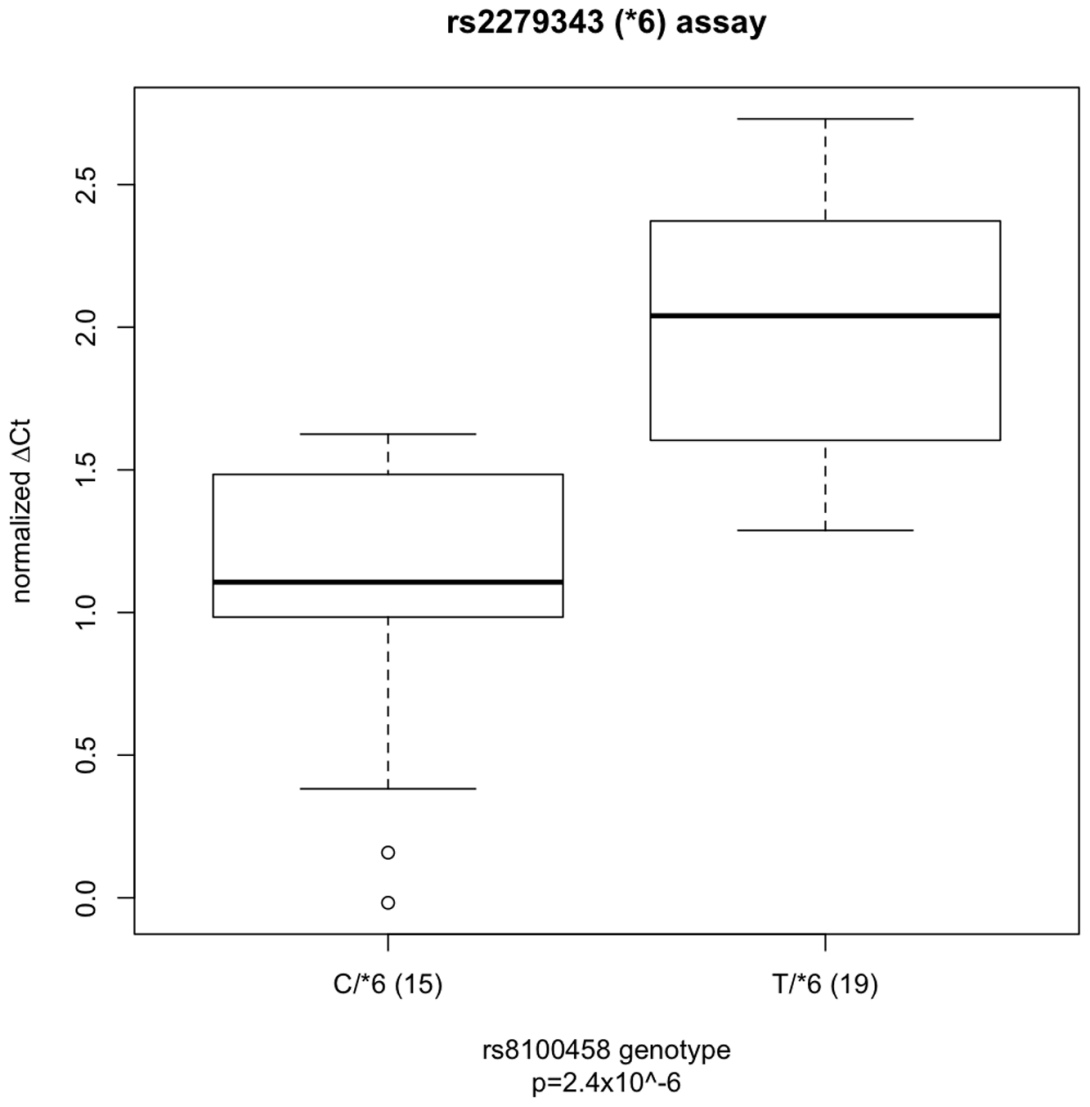
The ratio of allele-specific gene expression (ΔCt) for cDNAs from rs2279343 (CYP2B6*6) heterozygous liver biopsy samples. Samples, excluding rs3211371 heterozygotes, are either heterozygous (CT) at rs8100458 (C/*6, n = 15) or rs8100458 TT homozygotes (T/*6, n = 19). ΔCt differs significantly by genotype (p = 2.4×10^−6^). Relative expression, ΔCt, was determined by subtracting the smaller Ct value of one allele PCR reaction from the larger Ct value of the other allele PCR reaction normalized against the average ratio obtained from gDNAs for each genotype. The boxplot provides a summary of the data distribution. The box represents the interquartile range, which includes 50% of values. The line across the box indicates the median. The whisker lines extend to the highest and lowest values that are within 1.5x the interquartile range. Further outliers are marked with circles.

Ninety-nine European American liver samples were genotyped for rs8109525, rs8100458, rs3211371, rs3745274, rs2279343, and rs3786552. rs8109525 and rs8100458 were in perfect linkage disequilibrium (R^2^ = 1) in these samples. Because rs3211371, rs3745274, and rs2279343 are reported to be tightly linked to rs8109525/rs8100458 (D′ = 1 [36], R^2^<0.15, Figure 1), for the purpose of these analysis, CYP2B6*5 and *6 were assumed to be rs8109525/rs8100458 major allele haplotypes; this assumption could decrease our ability to detect real differences in allelic expression associated with rs8109525/rs8100458. In both sets of heterozygotes, using TaqMan assays for the SNPs rs3211371 and rs2279343 respectively, significantly different relative allelic expression was found between rs8109525/rs8100458 heterozygotes and homozygotes (for the rs3211371 assay, 1.09±0.13 vs. 0.52±0.38, p = 0.025, Figure 2; for rs2279343, 2.05±0.45 vs. 1.06±0.51, p = 2.4×10^−6^, Figure 3), consistent with lower expression of the minor allele haplotype and similar expression among different major allele haplotypes. Analyses of the rs2279343 assay data also find significant differences between rs3786552 major allele homozygotes and heterozygotes (data not shown). By comparison, using total expression assays we were not able to detect statistically significant differences in total *CYP2B6* expression predicted by any of these variants in this small sample (data not shown). Our results indicate differences in *CYP2B6* allelic expression that are associated with non-coding variation in the locus; this difference in expression provides a potential mechanism to explain why nicotine metabolism is associated with *CYP2B6* haplotype independent of coding variation in *CYP2A6* and *CYP2B6*.

### *CYP2B6* variants associated with aberrant splicing

**Figure 4:**
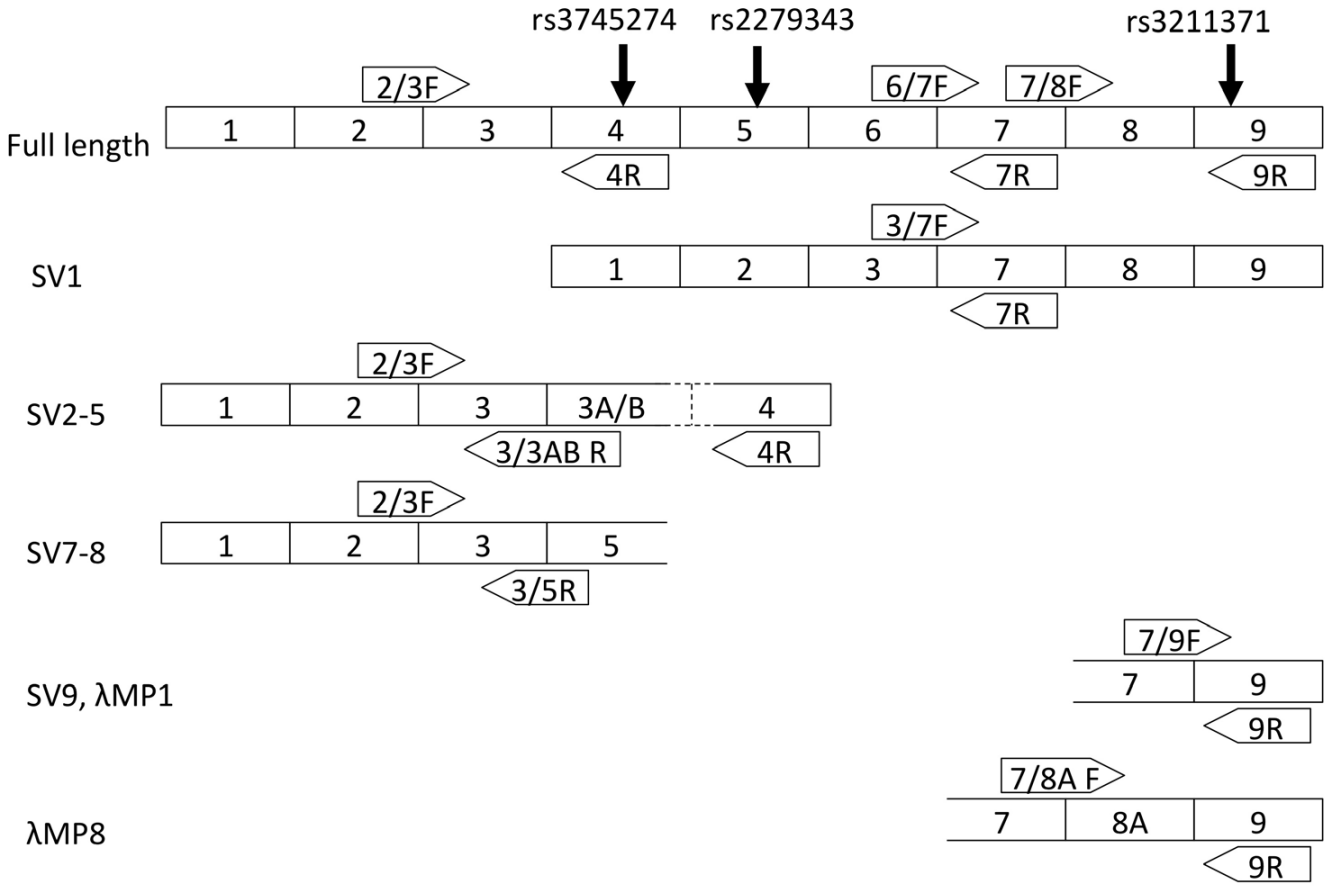
Common *CYP2B6* splice-forms, and the relative locations of primers used in quantitative real-time splice-form expression assays. Splice form nomenclature follows prior literature [27], [28], [38]. The locations of common non-synonymous variants are marked on the full-length transcript. Not to scale.

At least ten alternatively-spliced *CYP2B6* transcripts have been detected in human cDNAs, including a splice-form, SV1, associated with the CYP2B6*6 allele [27]. Allelic differences in expression might be due to differences in splicing efficiency and a preponderance of aberrantly-spliced transcripts produced by particular alleles. To determine the association between aberrant splicing and *CYP2B6* variants associated with nicotine metabolism or smoking cessation we chose to focus on five types of aberrant splicing previously detected in *CYP2B6*: 1) skipping exons 4–6 resulting in the splice-form called SV1; 2) inclusion of an additional exon (called 3A or 3B) between exons 3 and 4, resulting in splice-forms SV2, SV3, SV4 or SV5; 3) skipping exon 4 resulting in splice-forms SV7 or SV8; 4) skipping exon 8 resulting in splice-form SV9 or λMP1; and 5) inclusion of an alternative eighth exon (8A) resulting in a splice-form called λMP8 (Figure 4) [27], [28], [38]. With the exception of SV1, which lacks 160 amino acids including several in the active site but remains in frame, all of these alternative splicing events result in frame-shifts and premature stop codons. PCR primers were designed to cross exon splice junctions specific to different transcripts for quantitative real-time PCR assays. Stepwise regression analyses were performed on the different ratios of alternatively spliced transcripts (difference in PCR cycle time (ΔCT)) (Figure 4) using SNPs rs8100458, rs3786552, rs3211371 (CYP2B6*5), and rs3745274 (CYP2B6*6) as variables to determine those that optimally predict the relative concentration of alternatively spliced transcripts in the liver cDNAs.

**Figure 5:**
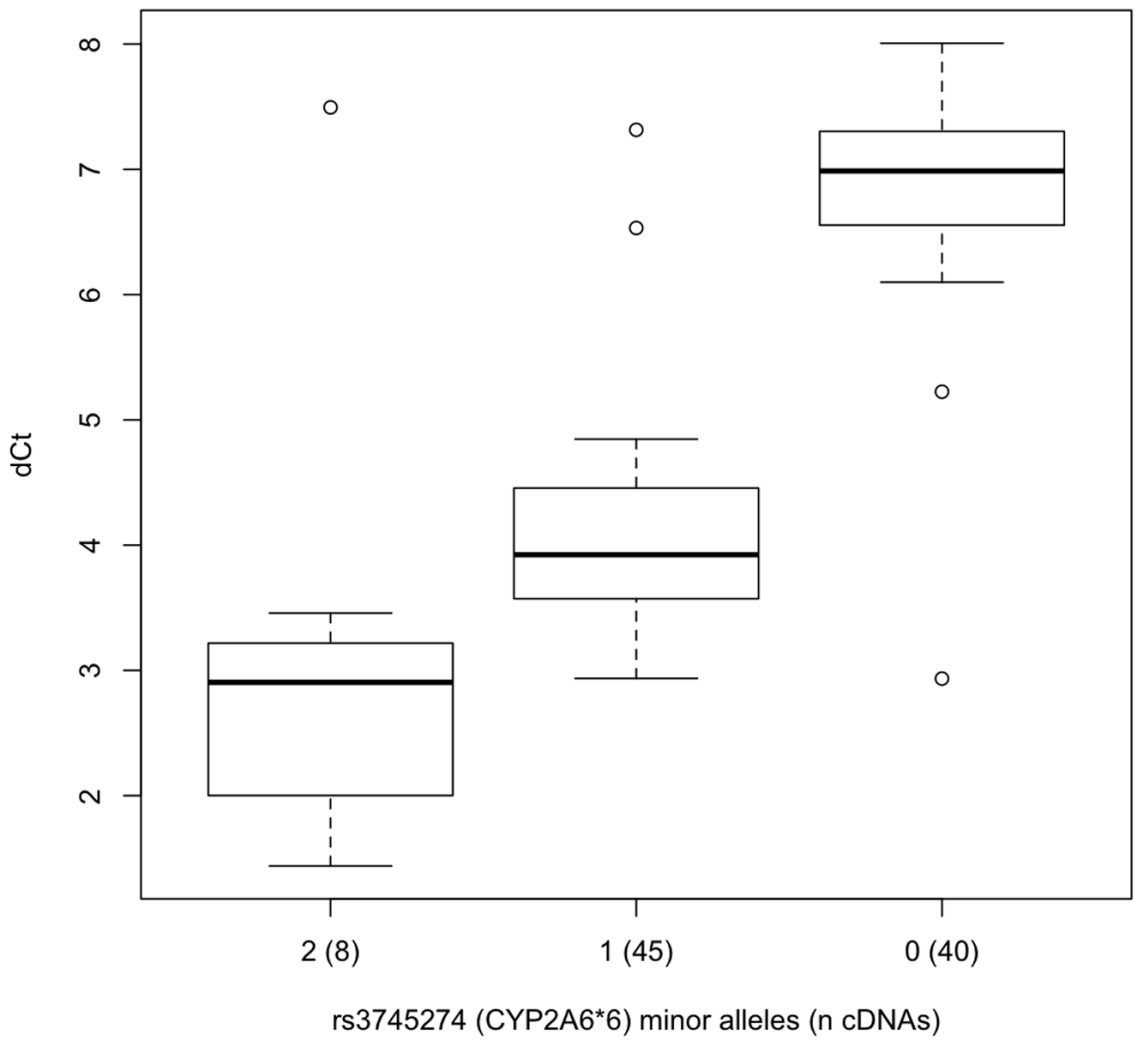
The ratio of aberrant exon 3-7 splicing (generating the SV1 splice-form) to correct exon 6-7 splicing. The difference in PCR cycle times (ΔCt) for cDNAs for (n) liver biopsy samples divided by rs3745274 (CYP2B6*6) genotype, as dictated by the optimum model predicting ΔCt (Table 3). Relative expression, ΔCt, was determined by subtracting the Ct value of the PCR reaction using primers ‘6/7F’ and ‘7R’ from the Ct value of the PCR reaction using primers ‘3/7F’ and ‘7R’ (Fig. 4). The boxplot provides a summary of the data distribution. The box represents the interquartile range, which includes 50% of values. The line across the box indicates the median. The whisker lines extend to the highest and lowest values that are within 1.5x the interquartile range. Further outliers are marked with circles.

**Figure 6:**
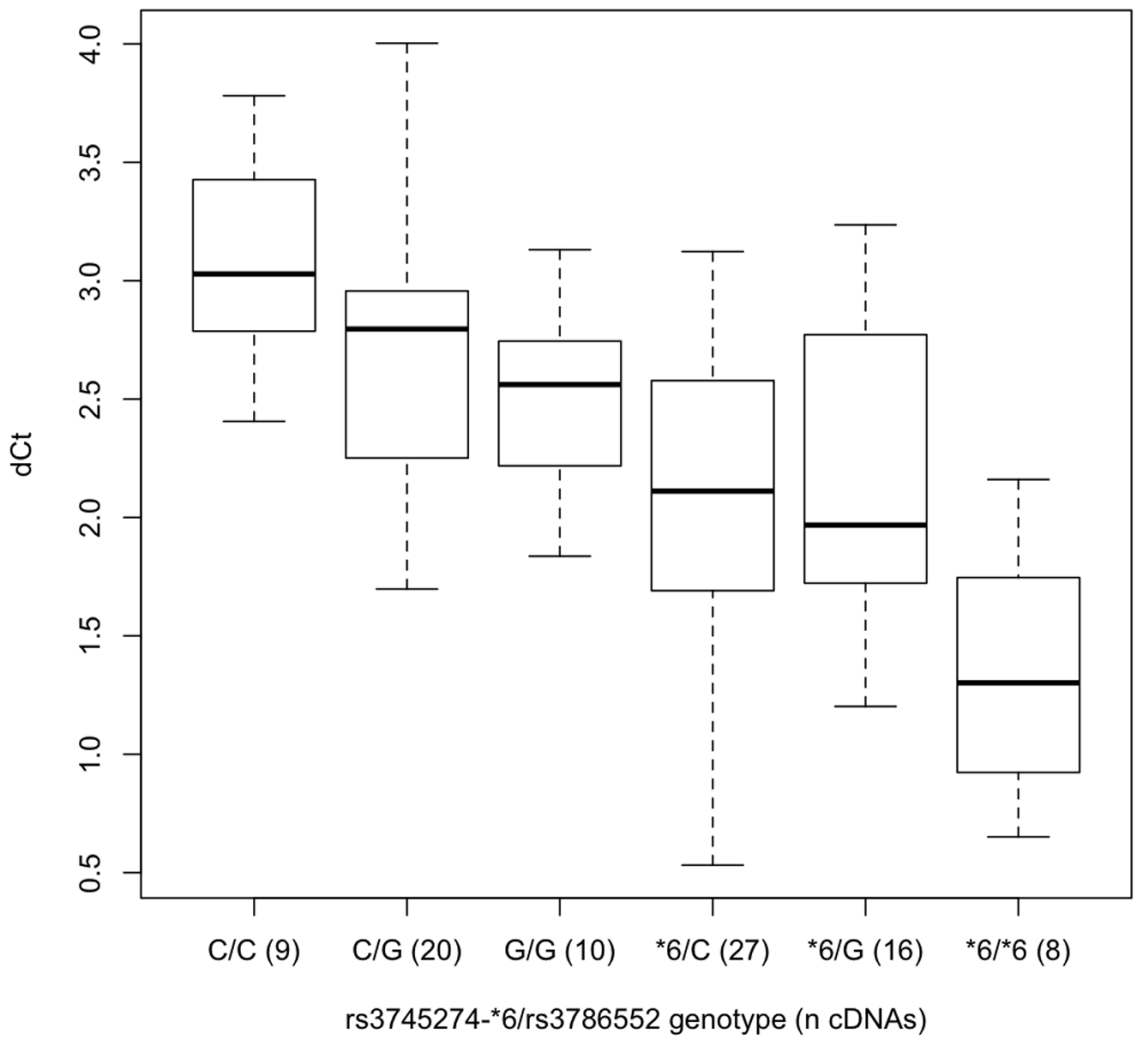
The ratio of aberrant exon 3-3A/B splicing (generating the SV2-5 splice-forms) to all transcripts that include exons 2, 3 & 4. The difference in PCR cycle times (ΔCt) for cDNAs for (n) liver biopsy samples divided by rs3745274 (CYP2B6*6) and rs3786552 genotype, as dictated by the optimum model predicting ΔCt (Table 4). Relative expression, ΔCt, was determined by subtracting the Ct value of the PCR reaction using primers ‘2/3F’ and ‘4R’ from the Ct value of the PCR reaction using primers ‘2/3F’ and ‘3/3AB R’ (Fig. 4). The boxplot provides a summary of the data distribution. The box represents the interquartile range, which includes 50% of values. The line across the box indicates the median. The whisker lines extend to the highest and lowest values that are within 1.5x the interquartile range. Further outliers are marked with circles.

**Figure 7:**
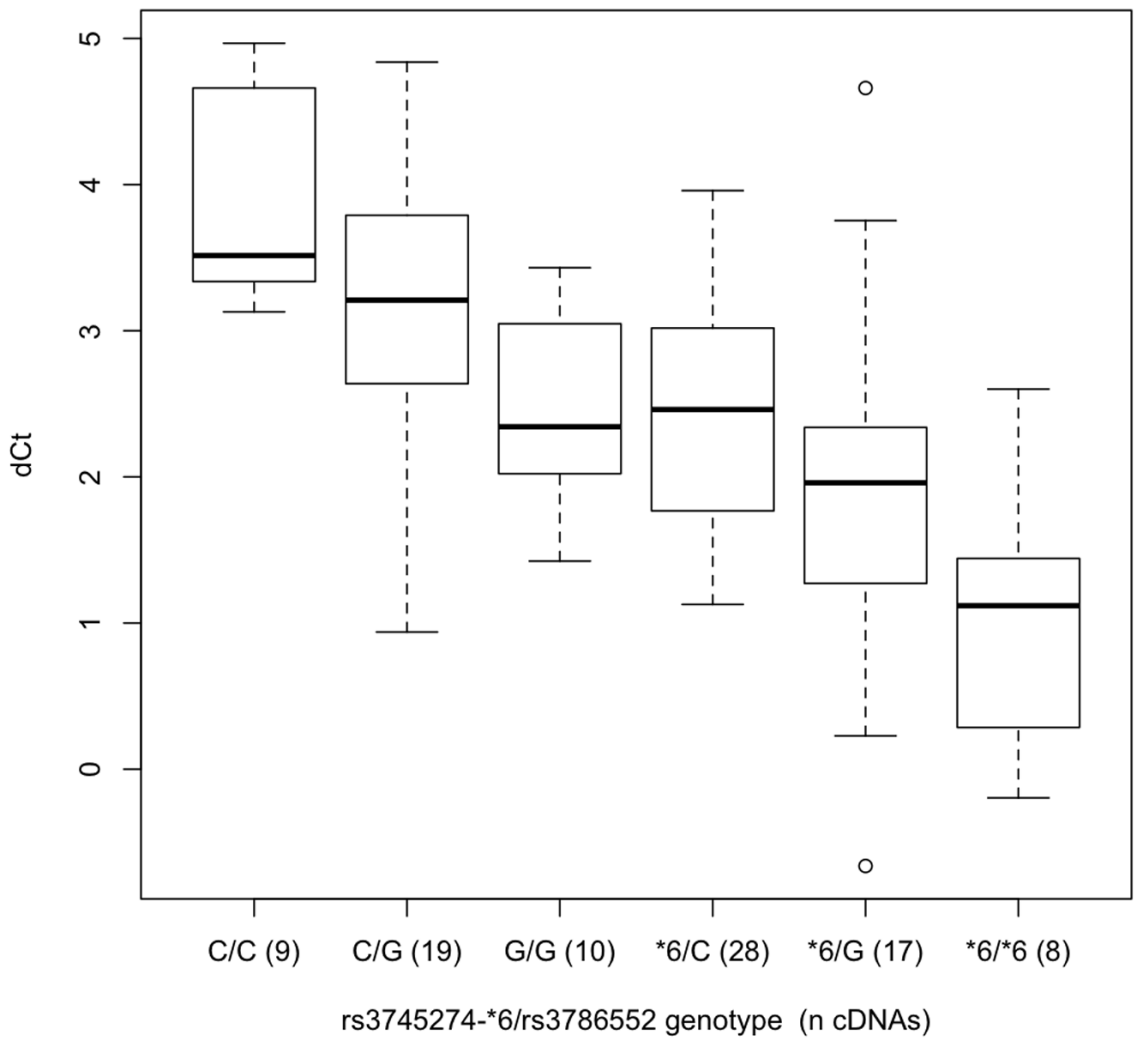
The ratio of aberrant exon 3-5 splicing (generating the SV7-8 splice-forms) to all transcripts that include exons 2, 3 & 4. The difference in PCR cycle times (ΔCt) for cDNAs for (n) liver biopsy samples divided by rs3745274 (CYP2B6*6) and rs3786552 genotype, as dictated by the optimum model predicting ΔCt (Table 5). Relative expression, ΔCt, was determined by subtracting the Ct value of the PCR reaction using primers ‘2/3F’ and ‘4R’ from the Ct value of the PCR reaction using primers ‘2/3F’ and ‘3/5R’ (Fig. 4). The boxplot provides a summary of the data distribution. The box represents the interquartile range, which includes 50% of values. The line across the box indicates the median. The whisker lines extend to the highest and lowest values that are within 1.5x the interquartile range.

**Figure 8:**
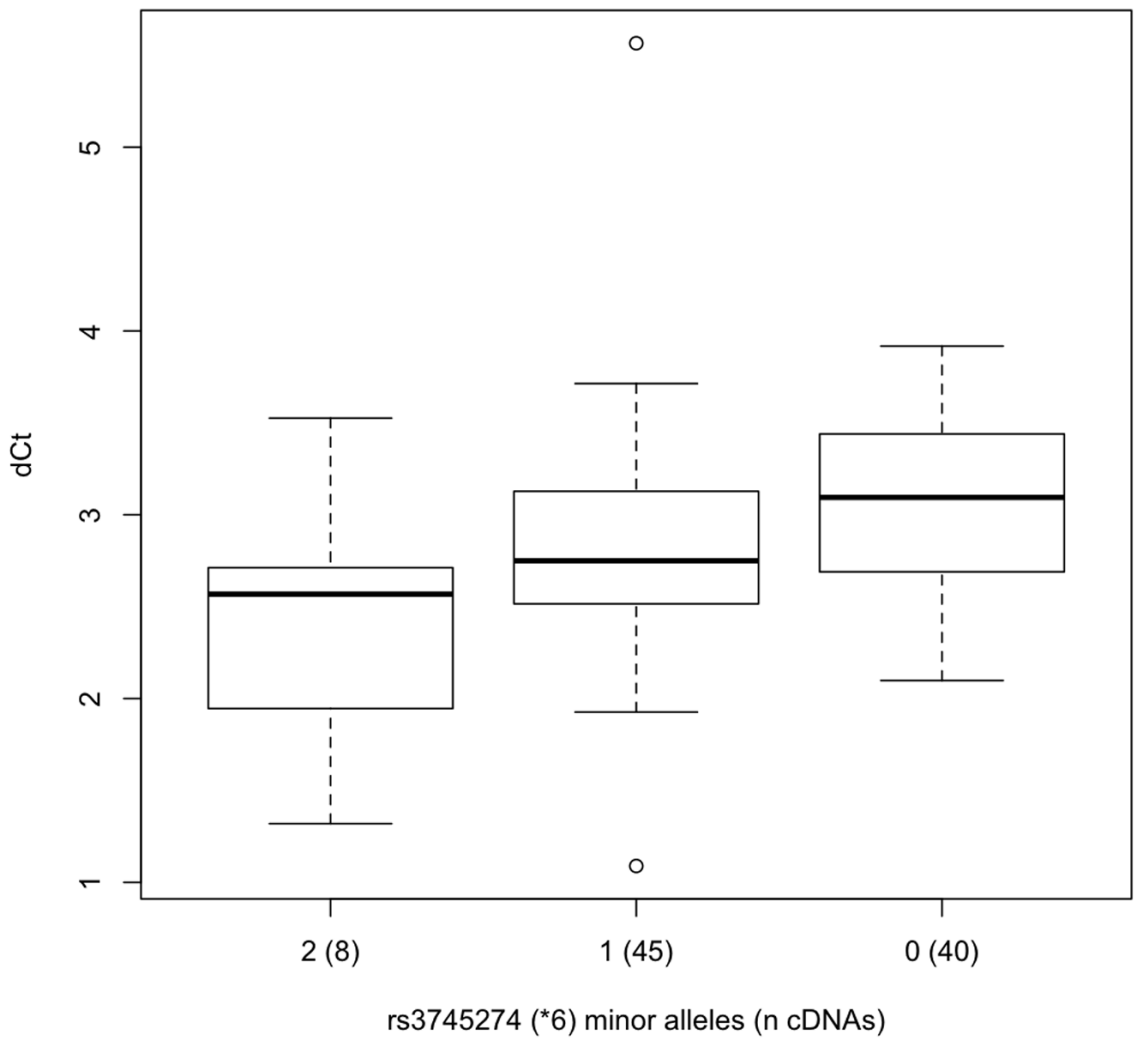
The ratio of aberrant exon 7-9 splicing (generating the SV9/λMP1 splice-forms) to correct exon 7-8 splicing. The difference in PCR cycle times (ΔCt) for cDNAs for (n) liver biopsy samples divided by rs3745274 (CYP2B6*6) genotype, as dictated by the optimum model predicting ΔCt (Table 6). Relative expression, ΔCt, was determined by subtracting the Ct value of the PCR reaction using primers ‘7/8F’ and ‘9R’ from the Ct value of the PCR reaction using primers ‘7/9F’ and ‘9R’ (Fig. 4). The boxplot provides a summary of the data distribution. The box represents the interquartile range, which includes 50% of values. The line across the box indicates the median. The whisker lines extend to the highest and lowest values that are within 1.5x the interquartile range. Further outliers are marked with circles.

**Figure 9:**
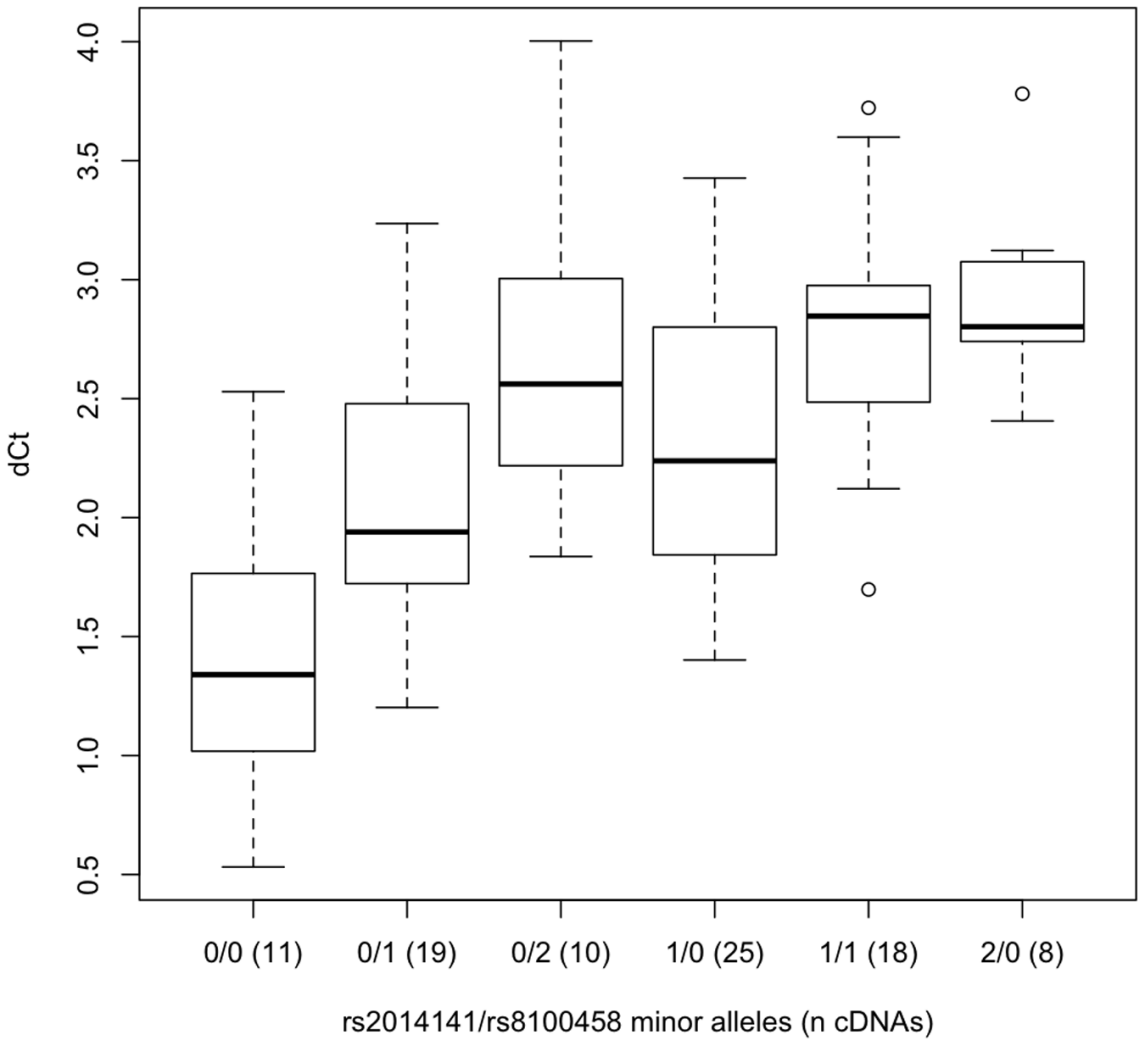
The ratio of aberrant exon 7-8A splicing (generating the λMP8 splice-form) to correct exon 7-8 splicing. The difference in PCR cycle times (ΔCt) for cDNAs for (n) liver biopsy samples divided by rs3745274 (CYP2B6*6) genotype, as dictated by the optimum model predicting ΔCt (Table 7). Relative expression, ΔCt, was determined by subtracting the Ct value of the PCR reaction using primers ‘7/8F’ and ‘9R’ from the Ct value of the PCR reaction using primers ‘7/8A F’ and ‘9R’ (Fig. 4). The boxplot provides a summary of the data distribution. The box represents the interquartile range, which includes 50% of values. The line across the box indicates the median. The whisker lines extend to the highest and lowest values that are within 1.5x the interquartile range. Further outliers are marked with circles.

Our results confirm that skipping of exons 4-6 resulting in SV1 is highly-significantly and specifically associated with the CYP2B6*6 allele (rs3745274, Table 3, Figure 5). SV1 is relatively common among CYP2B6*6 homozygotes—9:1 versus >110:1—i.e. transcripts containing the exon 6-7 splice junction outnumber those with aberrant exon 3-7 splice junction by ∼9:1 in CYP2B6*6 homozygotes (mean difference in PCR cycle time (ΔCt)  = 3.2, median = 2.9) compared to a ratio of >110:1 (mean ΔCt  = 6.8, median = 7.0) in non-CYP2B6*6 carriers (Figures 4 & 5). Other aberrant-splicing events appeared to be more common than SV1 across all genotypes, and were also significantly associated with *CYP2B6* genotype (Tables 4–7, Figures 6–9). The variant rs3211371, which defines the CYP2B6*5 allele, was not included in any optimum model that predicts aberrant splicing. Our results demonstrate a large potential range in expression of full-length *CYP2B6* transcript associated with different haplotypes.

**Table 3:** *CYP2B6* variants predicting aberrant exon 3−7 splicing (skipping exons 4−6), generating the SV1 splice-form, versus correct exon 6−7 splicing, individually or in an optimum multivariate model.

			multivariate model	
Variant	Individual R^2^	Individual p	aParameter estimate	p
rs8100458	0.14	1.2×10^−4^		
rs3786552	0.17	3.1×10^−5^		
rs3745274*6	0.66	4.1×10^−23^	−2.2±0.2	4.1×10^−23^
rs3211371*5	0.03	0.06		
Total adjusted R^2^ optimum model			0.66	

a Difference in PCR cycle time (ΔCt) determined by subtracting the Ct value of the PCR reaction using primers ‘6/7F’ and ‘7R’ from the Ct value of the PCR reaction using primers ‘3/7F’ and ‘7R’ (see Figure 4, primer sequences in table S4).

**Table 4:** *CYP2B6* variants predicting aberrant exon 3-3A/B splicing, generating the SV2-5 splice-forms, versus all transcripts that include exons 2, 3 & 4, individually or in an optimum multivariate model.

			multivariate model	
	Individual R^2^	Individual p	aParameter estimate	p
rs8100458	0	0.6		
rs3786552	0	0.5	−0.5±0.2	5.2×10^−3^
rs3745274*6	0.08	4.5×10^−3^	−0.8±0.2	8.6×10^−5^
rs3211371*5	0	0.3		
Total adjusted R^2^ optimum model			0.15	

a Difference in PCR cycle time (ΔCt) determined by subtracting the Ct value of the PCR reaction using primers ‘2/3F’ and ‘4R’ from the Ct value of the PCR reaction using primers ‘2/3F’ and ‘3/3AB R’ (see Figure 4, primer sequences in table S4).

**Table 5:** *CYP2B6* variants predicting aberrant exon 3-5 splicing, generating the SV7-8 splice-forms, versus all transcripts that include exons 2, 3 & 4, individually or in an optimum multivariate model.

			multivariate model	
	Individual R^2^	Individual p	aParameter estimate	p
rs8100458	0	0.9		
rs3786552	0	0.9	−0.7±0.2	1.1×10^−4^
rs3745274*6	0.28	3.3×10^−8^	−1.4±0.2	8.4×10^−12^
rs3211371*5	0.03	0.04		
Total adjusted R^2^ optimum model			0.40	

a Difference in PCR cycle time (ΔCt) determined by subtracting the Ct value of the PCR reaction using primers ‘2/3F’ and ‘4R’ from the Ct value of the PCR reaction using primers ‘2/3F’ and ‘3/5R’ (see Figure 4, primer sequences in table S4).

**Table 6:** *CYP2B6* variants predicting aberrant exon 7-9 splicing, generating the SV9 and λMP1 splice-forms, versus correct exon 7-8 splicing, individually or in an optimum multivariate model.

			multivariate model	
	Individual R^2^	Individual p	aParameter estimate	p
rs8100458	0.01	0.2		
rs3786552	0	0.2		
rs3745274*6	0.08	3.5×10^−3^	−0.3±0.1	3.5×10^−3^
rs3211371*5	0	0.3		
Total adjusted R^2^ optimum model			0.08	

a Difference in PCR cycle time (ΔCt) determined by subtracting the Ct value of the PCR reaction using primers ‘7/8F’ and ‘9R’ from the Ct value of the PCR reaction using primers ‘7/9F’ and ‘9R’ (see Figure 4, primer sequences in table S4).

**Table 7:** *CYP2B6* variants predicting aberrant exon 7-8A splicing, generating the λMP8 splice-form, versus correct exon 7-8 splicing, individually or in an optimum multivariate model.

			multivariate model	
	Individual R^2^	Individual p	aParameter estimate	p
rs8100458	0.04	0.03		
rs3786552	0.02	0.07		
rs3745274*6	0.34	6.5×10^−10^	−0.7±0.1	3.0×10^−10^
rs3211371*5	0.04	0.03		
Total adjusted R^2^ optimum model			0.34	

a Difference in PCR cycle time (ΔCt) determined by subtracting the Ct value of the PCR reaction using primers ‘7/8F’ and ‘9R’ from the Ct value of the PCR reaction using primers ‘7/8A F’ and ‘9R’ (see Figure 4, primer sequences in table S4).

## Discussion

Non-coding variants in the *CYP2B6* locus were recently identified as the most significant associations with smoking cessation in a candidate gene association study that included 785 SNPs in 24 genes [11]. The CYP2B6 enzyme has relatively little *in vitro* activity toward nicotine [8], but it is relevant to smoking-related phenotypes as the chief catalyst responsible for metabolism of the cessation drug bupropion. Intriguingly, the associations with cessation appeared to be independent of bupropion treatment. A possible solution to this mystery proposed by those authors [11] was that the SNPs were in high LD with functional variation in *CYP2A6*, the primary nicotine metabolism enzyme. *CYP2B6* and *CYP2A6* are located on chromosome 19 approximately 100 kilobases apart, suggesting that the non-coding variant identified might represent a ‘synthetic association’ i.e. a proxy SNP that joins through linkage disequilibrium multiple alleles, perhaps of both genes, thereby combining their effects upon bupropion and/or nicotine metabolism to result in the identified association with smoking cessation. However, contrary to this notion, we find that the variant is not closely linked to any functional variants in *CYP2A6*, and we show that non-coding variants in *CYP2B6* are significantly associated with differences in hepatic nicotine metabolism independent of coding variants in both *CYP2A6* and *CYP2B6*.

Prior investigations of genetic variation in *CYP2B6* have largely confined themselves to non-synonymous differences, focusing in particular on the common haplotype CYP2B6*6 which differs from the reference allele CYP2B6*1 by two amino acids (Q172>H and K262>R, table 1). Over forty publications in the last decade have addressed the question of CYP2B6*6 activity and have found its effect to be substrate specific; CYP2B6*6 is associated with slower metabolism of efavirenz [39]–[43] and methadone [44]–[46], but faster clearance of cyclophosphamide [47]–[49] and perhaps other substrates [50], [51] including nicotine and cotinine [34]. Reports have also confirmed opposite effects of the *6 variants on metabolism of efavirenz versus cyclophosphamide *in vitro* [52].

The mechanisms by which common polymorphisms in *CYP2B6* affect function remain poorly understood. The minor allele of the non-synonymous variant rs3745274 (Q172>H) is predicted to alter an exon splicing enhancer site [28]and Hofmann et al [27] demonstrated that it causes aberrant splicing of CYP2B6*6 transcripts to produce the SV1 splice-form lacking three internal exons. However, aberrant splicing cannot explain the apparent relative higher activity of CYP2B6*6 toward cyclophosphamide or nicotine [34]. Variation in splicing creates a special barrier to predicting gene function from genotype because it changes both the function and relative abundance of different transcripts. Aberrant *CYP2B6* splicing is very common and diverse and may be caused by many individual variants that interact with each other across the locus. The strong and specific association between the SV1 alternative splice-form and the *6 allele was previously reported and asserted as the key mechanism leading to altered *6 activity [27]. But our results indicate that aberrant production of SV1 may not be the primary contributor to reduced CYP2B6*6 expression. Unlike the prior study, our findings are based on comparison of assays amplifying PCR products of similar size (Figure S1) rather than comparing amplification of different products that include or skip three exons [27]. In fact, among the five aberrant splicing events assayed here that result in at least ten reported alternative splice-forms, all appear to be relatively common with the exception of SV1, which is rare in non-CYP2B6*6 carriers. Of course, quantifying transcript splicing in cDNAs cannot indicate the degree to which any particular alternative splice form displaces the expression of functional full-length transcript because of differences in perdurance of different splice-forms.

Our data also indicate differences in *CYP2B6* mRNA expression associated with genotype that cannot be straightforwardly explained by variation in alternative splicing. This variation appears to be associated with small differences in hepatic nicotine metabolism. rs8109525 and rs8100458, the key *CYP2B6* SNPs associated with smoking cessation [11] and nicotine metabolism, are also in high linkage disequilibrium (R^2^>0.98) with rs7254579 (-2320t>c) [36], a polymorphism predicted to disrupt a GATA transcription factor binding site [37]. This SNP and another, rs4802101 (called -750t>c [28]), predicted to disrupt an HNF-1α site, define three common classes of *CYP2B6* haplotypes previously described: *1A (TT), *1H/J (CC) and *6B (TC) (Table 1). rs7254579 and rs4803417 were each formerly investigated for their association with total *CYP2B6* expression yielding ambiguous results [37]. Given our results together with those of Hofmann et al [27], it is clear that the analysis of either SNP singly could be confounded by the consequences of the *6 allele or of other genetic variation upon splicing (i.e. exon skipping) depending on the targets of expression assay probes. The use of TaqMan allelic expression assays to determine the relative expression of two haplotypes in heterozygotes can partially ameliorate this problem by focusing experiments on particular haplotypes, as well as by correcting for the large amount of variance in total gene expression attributable to non-genetic factors. We were able to assay two SNPs, rs2279343 and rs3211371, located in exons 5 and 9 respectively. Both variants therefore occur in all identified alternative splice forms with the exception of SV1. Results from both assays indicate that *1A is more highly expressed than *1H/*1J, consistent with other published expression data [37] and parallel to the relatively large but not statistically significant difference reported by Hofmann et al [27]. These results indicate that there are genetically determined differences in *CYP2B6* expression that are not explained by differences in splicing.

Our results demonstrate that common functional variation in the *CYP2B6* locus does not begin and end with CYP2B6*6. Ultimately, we must conclude that variation across the *CYP2B6* locus influences expression and splicing that may lead to differences in *in vivo* CYP2B6 activity which are however impossible to predict from *in vitro* results alone. These differences may account for previous contradictory reports regarding the influence of *CYP2B6* genotype upon bupropion metabolism [53], [54] or smoking cessation [55]–[58]. Fortunately, associations between genotype and function can be examined without first fully elaborating the molecular mechanisms underlying the functional differences. In a recent *in vivo* study of nicotine metabolism we conducted in ∼200 subjects we were able to define activities of different common *CYP2A6* haplotypes with high confidence based on few assumptions about the functional effects of the variants [30]. In multiple instances those *in vivo* results forced a reevaluation of assumptions about specific alleles that had been made based on *in vitro* experiments. Those findings also indicated previously unrecognized differences between alleles that we subsequently determined to be associated with mRNA splicing efficiency [24]. In the case of *CYP2B6*, much of the common variation would appear to affect gene transcription or splicing, and these are not likely to be substrate specific. These discrepancies could be resolved using a similar *in vivo* metabolism experiment with an appropriate CYP2B6 substrate, a sufficient number of subjects, and most importantly, thorough determination of *CYP2B6* haplotypes. Such comprehensive results may then allow us to retrospectively understand how the diversity of variation in expression, splicing, and enzyme activity collaborate to determine the relative impacts of different common *CYP2B6* alleles on metabolism and smoking cessation.

## Supporting Information

Figure S1 **Splicing primer products from liver cDNAs.**
(TIFF)

Table S1 **Characteristics of COGEND metabolism experiment subjects.**
(DOCX)

Table S2 **Determining** ***CYP2A6*** **diplotype and predicted metabolism metric from gene copy number and 6 SNPs.**
(DOCX)

Table S3 **Genotyping primers. Splicing primers.**
(DOCX)
