# Supplementary figures and images for: CYP2B6 Non-Coding Variation Associated with Smoking Cessation Is Also Associated with Differences in Allelic Expression, Splicing, and Nicotine Metabolism Independent of Common Amino-Acid Changes
Source: PLoS One. 2013 Nov 15;8(11):e79700. doi: 10.1371/journal.pone.0079700 (PMC3829832; doi:10.1371/journal.pone.0079700)

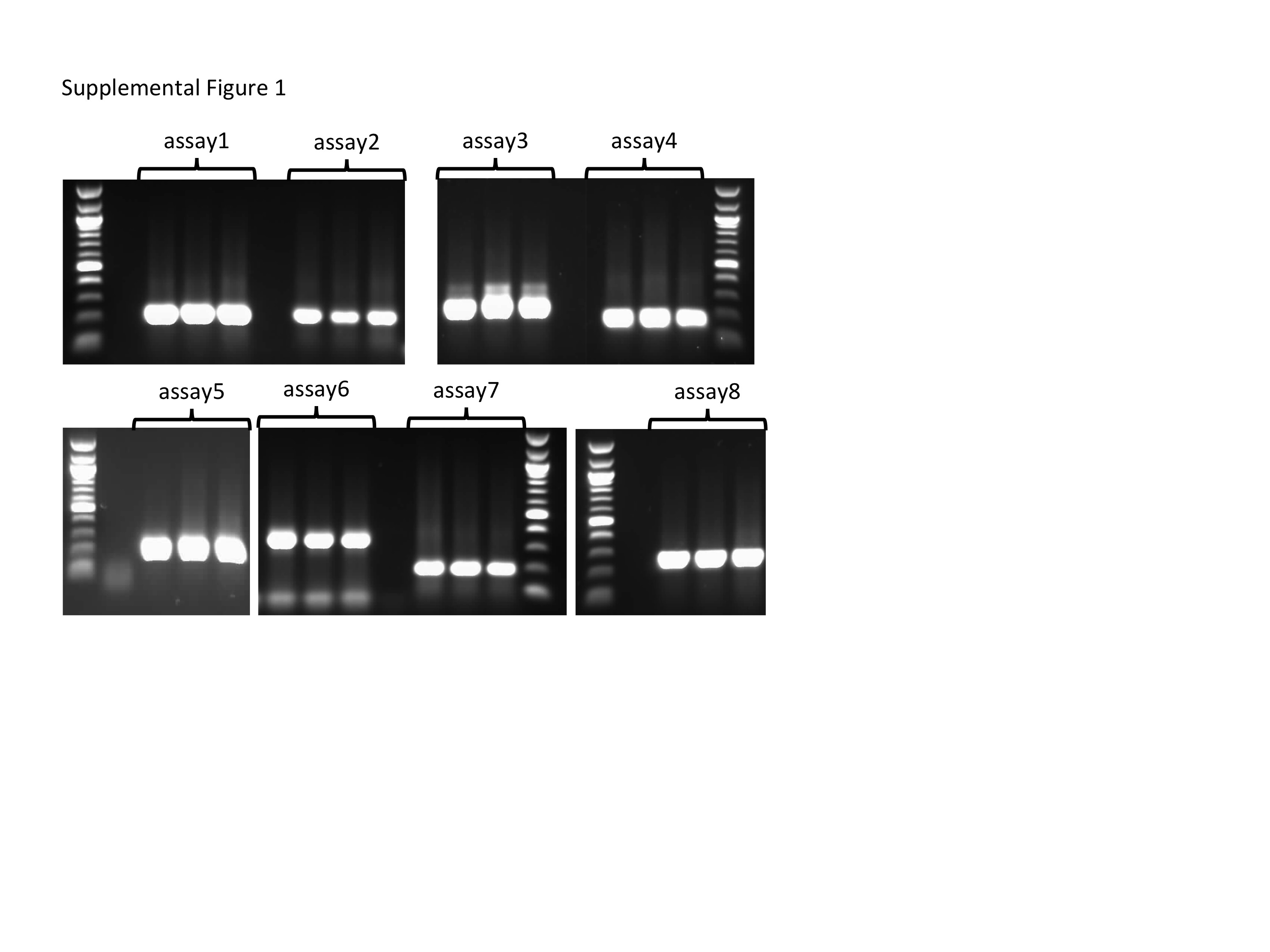

Supplement: Figure S1 — Splicing primer products from liver cDNAs. (TIFF) [file pone.0079700.s001.tiff]
