# Supplementary material for: CYP2B6 Non-Coding Variation Associated with Smoking Cessation Is Also Associated with Differences in Allelic Expression, Splicing, and Nicotine Metabolism Independent of Common Amino-Acid Changes
Source: PLoS One. 2013 Nov 15;8(11):e79700. doi: 10.1371/journal.pone.0079700 (PMC3829832; doi:10.1371/journal.pone.0079700)
Supplement: Table S1 — Characteristics of COGEND metabolism experiment subjects. (DOCX) [file pone.0079700.s002.docx]

Supplemental Table 1. Characteristics of COGEND metabolism experiment subjects

| Category | n | mean age + SD (yrs) | mean weight + SD (lbs) | mean body mass index + SD |
| --- | --- | --- | --- | --- |
| Male current smoker | 52 | 37.1 + 5.5 | 202+53 | 27.8+6.4 |
| Male former smoker | 36 | 37.5 + 4.3 | 202+45 | 27.9+5.5 |
| Female current smoker | 50 | 35.5 + 5.3 | 154+37 | 25.8+6.3 |
| Female former smoker | 50 | 38.2 + 4.6 | 152+25 | 25.2+4.6 |

Current smoking status defined by a mean non-deuterated cotinine measurement of >2 ng/ml. One male subject using a nicotine patch is excluded.
