# Supplementary material for: CYP2B6 Non-Coding Variation Associated with Smoking Cessation Is Also Associated with Differences in Allelic Expression, Splicing, and Nicotine Metabolism Independent of Common Amino-Acid Changes
Source: PLoS One. 2013 Nov 15;8(11):e79700. doi: 10.1371/journal.pone.0079700 (PMC3829832; doi:10.1371/journal.pone.0079700)
Supplement: Table S2 — Determining CYP2A6 diplotype and predicted metabolism metric from gene copy number and 6 SNPs. (DOCX) [file pone.0079700.s003.docx]

**Supplemental Table 2. Determining *CYP2A6* diplotype and predicted metabolism metric from gene copy number and 6 SNPs**

| *CYP2A6* Copy number | rs1801272+ rs28399442+ rs148166815 | rs28399433 | rs1137115 | rs28399435 | Haplotye 1 | Haplotye 2 | Predicted Metabolism Metric |
| --- | --- | --- | --- | --- | --- | --- | --- |
| 0 |  | | | | *4 | *4 | 0.44 |
| 1 | ≥1 |  | | | *4 | *2/*4/*12/*38 | 0.44 |
| ≥2 | 2 |  | | | *2/*4/*12/*38 | *2/*4/*12/*38 | 0.44 |
|  | 1 | ≥1 |  | | *2/*4/*12/*38 | *9 | 0.64 |
|  |  | 0 | ≥1 | 0 | *2/*4/*12/*38 | *1A | 0.68 |
|  |  |  |  | ≥1 | *2/*4/*12/*38 | *14 | 0.76 |
|  |  |  | 0 |  | *2/*4/*12/*38 | Other | 0.76 |
|  | 0 | 2 |  | | *9 | *9 | 0.76 |
|  |  | 1 | ≥1 | 0 | *9 | *1A | 0.79 |
|  |  |  |  | ≥1 | *9 | *14 | 0.85 |
|  |  |  | 0 |  | *9 | Other | 0.85 |
|  |  | 0 | 2 | 0 | *1A | *1A | 0.82 |
|  |  |  |  | 1 | *1A | *14 | 0.87 |
|  |  |  |  | 2 | *14 | *14 | 0.90 |
|  |  |  | 1 | 0 | *1A | Other | 0.87 |
|  |  |  |  | ≥1 | Other | *14 | 0.90 |
|  |  |  | 0 |  | Other | Other | 0.90 |

Decision Matrix Progresses from left to right

rs1801272 TT=0, TA=1, AA=2

rs28399442 CC=0, CA=1, AA=2

rs148166815 AA=0, AG=1, GG=2

rs28399433 TT=0, TG=1, GG=2

rs1137115 GG=0, GA=1, AA=2
