# Supplementary material for: CYP2B6 Non-Coding Variation Associated with Smoking Cessation Is Also Associated with Differences in Allelic Expression, Splicing, and Nicotine Metabolism Independent of Common Amino-Acid Changes
Source: PLoS One. 2013 Nov 15;8(11):e79700. doi: 10.1371/journal.pone.0079700 (PMC3829832; doi:10.1371/journal.pone.0079700)
Supplement: Table S3 — Genotyping primers. Splicing primers. (DOCX) [file pone.0079700.s004.docx]

Supplemental Table 3

| rs12721655 | A allele | GAAGGTGACCAAGTTCATGCTATGAGGGACTTCGGGATGGGAA |
| --- | --- | --- |
|  | G allele | GAAGGTCGGAGTCAACGGATTGAGGGACTTCGGGATGGGAG |
|  | Common | TCCGCTCCTCCACACTCYGCT |
| rs2279343 | A allele | GAAGGTGACCAAGTTCATGCTAGGTAGGTGTCGATGAGGTCCT |
|  | G allele | GAAGGTCGGAGTCAACGGATTGGTAGGTGTCGATGAGGTCCC |
|  | Common | GCTTAYATTGGCCACAGTGTGGAGAA |
| rs28399499 | C allele | GAAGGTGACCAAGTTCATGCTGTGGGCCAATCACCTGTTCAG |
|  | T allele | GAAGGTCGGAGTCAACGGATTATGTGGGCCAATCACCTGTTCAA |
|  | Common | TGCCTCTTTMAAATGAGATTCATTGGTCTT |
| rs3211371 | C allele | GAAGGTGACCAAGTTCATGCTAAAATACCCCCAACATACCAGATCC |
|  | T allele | GAAGGTCGGAGTCAACGGATTCAAAATACCCCCAACATACCAGATCT |
|  | Common | ACCCTGGAATYCTTTGACCCCCTT |
| rs34223104 | C allele | GAAGGTGACCAAGTTCATGCTGCCTCTGCACCCTGTTATG |
|  | T allele | GAAGGTCGGAGTCAACGGATTCCTGCCTCTGCACCCTGTTATA |
|  | Common | GATGATGATGAAAAAGGAGGTGGGGAA |
| rs35303484 | A allele | GAAGGTGACCAAGTTCATGCTGATTTGAGTAGGCCTCTTCTATCCAT |
|  | G allele | GAAGGTCGGAGTCAACGGATTTGAGTAGGCCTCTTCTATCCAC |
|  | Common | GCCCTCTGCCCCTTTTGGGAAA |
| rs35979566 | A allele | GAAGGTGACCAAGTTCATGCTCAGGACACAGAAGTATTTCTCAA |
|  | T allele | GAAGGTCGGAGTCAACGGATTCTCAGGACACAGAAGTATTTCTCAT |
|  | Common | AAGTAGTGTGGGTCAYGGAGAGCA |
| rs36060847 | A allele | GAAGGTGACCAAGTTCATGCTAATGGGTCGACCATGGCGATTTTTT |
|  | G allele | GAAGGTCGGAGTCAACGGATTGGGTCGACCATGGCGATTTTTC |
|  | Common | TACGGGAGGCCCTTGTGGACAA |
| rs3745274 | G allele | GAAGGTGACCAAGTTCATGCTAGATGATGTTGGCGGTAATGGAC |
|  | T allele | GAAGGTCGGAGTCAACGGATTCAGATGATGTTGGCGGTAATGGAA |
|  | Common | CCAGGCACTTCAGTCTGTGTCSTT |
| rs8109525 | A allele | GAAGGTGACCAAGTTCATGCTGTTTGGGAAAGACTTTATTTCCTTTTCAT |
|  | G allele | GAAGGTCGGAGTCAACGGATTGTTTGGGAAAGACTTTATTTCCTTTTCAC |
|  | Common | YTGCAAGATACTGTACCCAGAAAAGTTATA |
| rs8100458 | C allele | GAAGGTGACCAAGTTCATGCTCACGAGTGCCTACCTTGAGTG |
|  | T allele | GAAGGTCGGAGTCAACGGATTCCACGAGTGCCTACCTTGAGTA |
|  | Common | GACCTGTGAGGAGAAAGAACAGAGTA |
| rs8192709 | C allele | GAAGGTGACCAAGTTCATGCTCTTGCTACTCCTGGTTCAGC |
|  | T allele | GAAGGTCGGAGTCAACGGATTACTCTTGCTACTCCTGGTTCAGT |
|  | Common | CCCTGGTGGGAGGCGGTCA |
